# Sentiment Classification for Financial Texts Based on Deep Learning

**DOI:** 10.1155/2021/9524705

**Published:** 2021-10-11

**Authors:** Shanshan Dong, Chang Liu

**Affiliations:** ^1^Department of Economics in Engineering and Technology College, Hubei University of Technology, Wuhan, Hubei 432200, China; ^2^Department of Qualitative Economics and Mathematics, School of Statistics and Mathematics, Zhongnan University of Economics and Law, Wuhan, Hubei 430073, China

## Abstract

Sentiment classification for financial texts is of great importance for predicting stock markets and financial crises. At present, with the popularity of applications in the field of natural language processing (NLP) adopting deep learning, the application of automatic text classification and text-based sentiment classification has become more and more extensive. However, in the field of financial text-based sentiment classification, due to a lack of labeled samples, such applications are limited. A domain-adaptation-based financial text sentiment classification method is proposed in this paper, which can adopt source domain (SD) text data with sentiment labels and a large amount of unlabeled target domain (TD) financial text data as training samples for the proposed neural network. The proposed method is a cross-domain transfer-learning-based method. The domain classification subnetwork is added to the original neural network, and the domain classification loss function is also added to the original training loss function. Therefore, the network can simultaneously adapt to the target domain and then accomplish the classification task. The experiment of the proposed sentiment classification transfer learning method is carried out through an open-source dataset. The proposed method in this paper uses the reviews of Amazon Books, DVDs, electronics, and kitchen appliances as the source domain for cross-domain learning, and the classification accuracy rates can reach 65.0%, 61.2%, 61.6%, and 66.3%, respectively. Compared with nontransfer learning, the classification accuracy rate has improved by 11.0%, 7.6%, 11.4%, and 13.4%, respectively.

## 1. Introduction

With the rapid development of technologies such as big data, artificial intelligence, and deep learning, how to adopt these technologies to the financial field has become a research hotspot. The sentiment classification of financial texts based on machine learning is of great significance for predicting stock markets and financial crises. Compared with traditional methods based on financial text sentiment classification, it has the following advantages: (1) the influence of human emotional factors can be reduced and (2) a large amount of data samples can be adopted for analysis. As a matter of fact, sentiment prediction of financial text based on machine learning has become a research hotspot. Through the analysis of current financial texts from the Internet, a preliminary judgment can be made about current economic fundamentals.

Currently, there has been in-depth research in the field of text classification. In terms of text representation, Collobert [1] firstly proposed to represent the text through the word vectors. References [2, 3] proposed a word2vec representation method. In this method, words not only can be represented by vectors, but the representation also embeds the word vectors to another space so that the vector distance in this space can express semantic and syntactic similarity. The representation method of word2vec has adopted the prior information obtained in the corpus, which is trained through the CBOW model [4] or skip-gram [5] model to obtain the vector representation of words. Generally speaking, since the corpus covers a wide range of texts, the mentioned word representation method is also representative. Reference [6] has proposed the doc2vec model by extending words to paragraphs, where the granularity of the text representation is changed. In terms of text-based classification, it can be divided into traditional machine-learning-based and deep-learning-based methods. The authors in [7] proposed a method of hierarchical support vector machine (SVM) to classify texts. The authors in [8] proposed to improve the pruning strategy of the traditional decision tree model to classify the text, which has achieved good results. The authors in [9] proposed to use the Naive Bayes model to classify text. Since the deep-learning-based methods can extract more abstract and higher-level features from the samples, they generally have better classification performances than traditional methods. The authors in [10] firstly introduced deep learning into the field of natural language processing (NLP). The authors in [11] combine word vectors and convolutional neural networks for the sentiment classification of texts and has achieved good results.

The topic of this paper is sentiment classification for financial texts. Judging whether the financial text is positive through the classification network can be of great significance for judging the current economic situations or investment enthusiasm. It has great significance for predicting the stock market and financial crisis. However, in the field of financial text sentiment classification, the biggest difference from text sentiment classification in other domains is the lack of a large number of labeled samples. Therefore, the application of sentiment classification in the financial text has been greatly restricted. In order to adopt deep learning methods for performing sentiment analysis on financial texts, a straightforward idea is to use cross-domain learning methods to transfer sentiment classification knowledge from other domains to the domain of financial texts. Generally speaking, the methods of transfer learning in this field can be divided into three categories. (1) The first category is the parameter fine-tuning-based method. In [12], by pretraining the convolutional neural network using the source domain data and then adopting the target domain data to fine-tune the parameters of the neural network, the original neural network can be transferred to the target domain. One disadvantage of this method is that it requires a large amount of labeled target domain data. Since the amount of labeled data in financial text is not sufficient, this method is not suitable for the application of financial text classification. The authors in [13] propose to freeze shallow network parameters in the process of parameter fine-tuning and only change higher-layer network parameters. The principle for the method is that the shallow features of cross-domain samples are the same, but the abstract features in the high level are different. Therefore, only high-level features need to be transferred. This method can reduce the number of labeled samples needed in the target domains but is still not suitable for the application in our paper. (2) The second category is feature representation learning methods. The authors in [14, 15] propose that cross-domain representation learning can be performed through stacked denoising autoencoder (SDA). After learning, the feature extraction neural network can be obtained. The network can extract shared features between different data domains. By adopting the extracted shared feature vectors, the problem of cross-domain text sentiment classification can be solved through the SVM classifier. In this method, no labeled target domain samples are required. The authors in [16, 17] propose a method for financial text sentiment classification based on a generative confrontation network (GAN), which combines the random noise generated in the generative network with text representation vectors. Then a discriminant network module is adopted to distinguish true source domain samples, generated samples, and the sentiment. This method can achieve good results, but the training of the network need large-volume datasets. The authors in [18, 19] propose to adopt the active learning method, which can effectively reduce the number of labeled samples in the target domain. The authors in [20, 21] propose a method of adversarial learning; by sentiment classification learning and domain discrimination learning, the purpose of domain adaptation can be achieved.

A financial text sentiment classification method based on unsupervised domain adaptation (DA) is proposed in this paper. The proposed method can transfer the sentiment classification knowledge of the source domain to the target domain of the financial text. Although the distribution of the source domain and the target domain are different, the shared features can still be learned for classification. The proposed method has the following advantages: (1) it does not require labeled samples in financial texts, so the method is unsupervised and suitable for the actual situation of lack of sentiment labels in financial texts and (2) the network structure adopted in the proposed method is similar to the original network structure, where the only difference is that the domain classification subnetwork needs to be added. This can make the proposed network structure less complex. The proposed method is a cross-domain neural network transfer learning method. The domain classification subnetwork is added to the traditional sentiment classification network, and the domain classification cost is added to the original training cost so that the network can simultaneously adapt to the target domain as well as the source domain. The experiment of emotion classification migration learning is carried out through the open-source dataset. The experiment of the proposed sentiment classification transfer learning method is carried out through the open-source dataset. The proposed method in this paper uses the reviews of Amazon Books, DVDs, electronics, and kitchen appliances as the source domain for cross-domain learning, and the classification accuracy rates have reached 65.0%, 61.2%, 61.6%, and 66.3%. Compared with nontransfer learning, the classification accuracy rate has improved by 11.0%, 7.6%, 11.4%, and 13.4%, respectively.

## 2. Methods

The problem to be studied in this paper can be expressed as follows: after training with labeled text data in the source domain and unlabeled data in the target domain, emotion classification is performed on the target domain, that is, financial text. Among them, the vector of the text sample of the source domain is represented as **x**_*i*_, where *i* represents the vector representation of the *i*-th sample, and the corresponding label can be represented as *s*_*i*_, where *i* represents the label of the *i*-th sample. For the target domain sample, it is represented as **y**_*i*_, and the corresponding domain label is *d*_*i*_. In the application of this article, the number of classifications required is 2, which are positive evaluation labels and negative evaluation labels. In this method, the model is trained according to the source domain samples and part of the target domain sample set, and then the target domain sample test set is used to test the accuracy of the model. In this section, the model is introduced according to the following parts. First, the overall model of domain adaptation in this article is introduced. Second, the cost function of the model in this article is introduced. Finally, the training process of the proposed model is introduced.

### 2.1. The Structure of the Proposed Network

**Figure 1:**
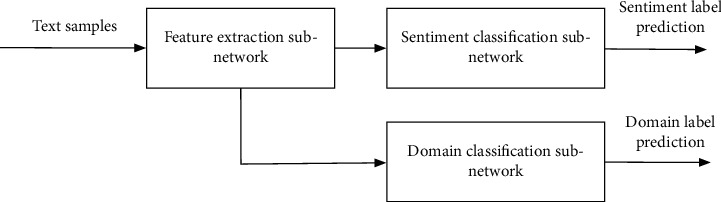
The network model of the proposed method.

The proposed model in this paper is shown in Figure 1. It can be seen that the model in this paper has added a domain classification subnetwork to the traditional classification model. The idea of domain transfer learning herein is: through the addition of domain classification subnetwork, the extracted features cannot distinguish the data domains. As a result, the extracted features from different data domains are more similar, thus achieving the transfer learning purpose.

**Figure 2:**
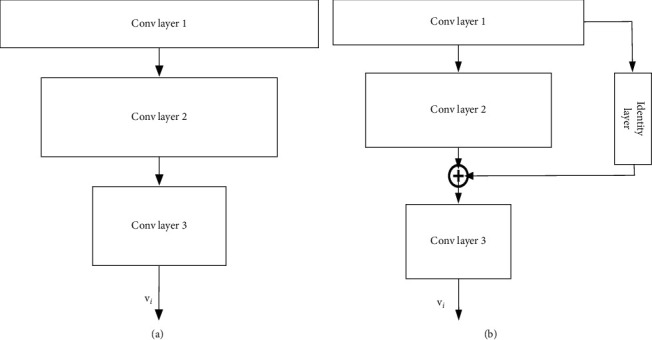
The detailed structure of the feature extraction subnetwork: (a) convolutional network and (b) residual network.

**Figure 3:**
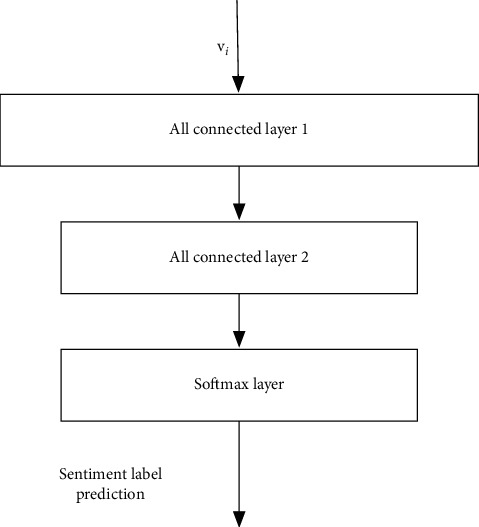
The sentiment classification subnetwork structure.

**Figure 4:**
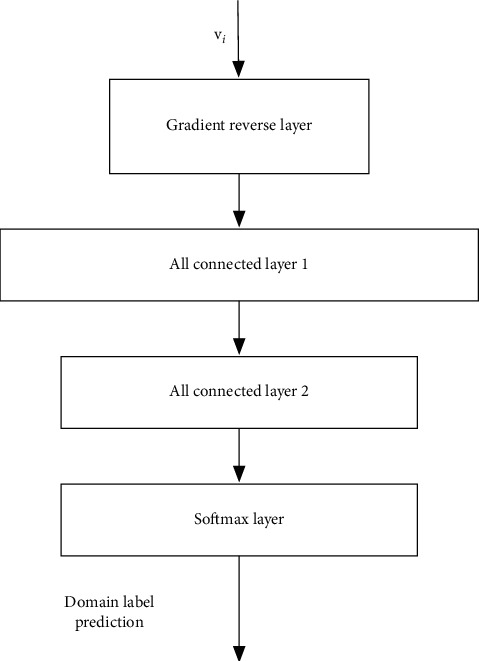
The domain classification subnetwork structure.

The network structure proposed in this paper is composed of three substructures: feature extraction subnetwork, sentiment prediction subnetwork, and domain classification subnetwork. The details of the three subnetworks are introduced as follows:

(1) The detailed structure of the feature extraction subnetwork is shown in Figure 2. The output is the extracted feature vector, which can be denoted as **v**_*i*_. The feature extraction network in this paper can have two structures. As shown in the figure, the first one is a common convolutional network structure, including three layers of convolutional network layers. The feature extraction subnetwork is similar to the traditional convolutional network layers, which extracts from shallow to deep features in the samples for classification. The second one is the feature extraction structure from a residual network. Since the residual network has direct links between convolutional layers that are not adjacent, it is usually easier to train. In our implementation, the residual network is adopted as the structure for the feature extraction subnetwork. In traditional convolutional neural networks, since there is only the cost function for classification, the extracted features can be used to distinguish sample labels. However, herein, the sentiment classification cost and the domain classification cost are both adopted. Through training, the feature extraction subnetwork can extract features that meet the following two conditions: firstly, the features can be effectively adopted for sentiment differentiation, and secondly, it is impossible to distinguish which domain the input sample comes from according to the extracted features, for example, the data domain of financial text or ordinary product review text.
(2) The structure of the sentiment classification subnetwork is shown in Figure 3, which consists of two fully connected layers plus a softmax layer for the final sentiment prediction. The output features of the previous feature extraction subnetwork can be adopted as the input, and the output denotes the probability of whether the sample belongs to the positive evaluation or not. The feature extraction subnetwork and sentiment classification subnetwork mentioned above can form a traditional sentiment classification neural network. Therefore, in this paper, the existing neural network structure of sentiment classification can be directly used. Herein, a cross-domain sentiment classification network can be obtained with improvement on the existing sentiment classification network. The following equation denotes the softmax layer operation, where *N* represents the number of categories and *e*^*i*^ represents the feature input of the dimension *i*.
  (1) $$\begin{array}{c} O_{i}=\frac{e^{i}}{\sum_{j=1}^{N}e^{j}}. \end{array}$$
(3) The structure of the domain classification network is shown in Figure 4. For the domain classification subnetwork, its main purpose is to be able to distinguish whether the samples come from the source domain or the target domain (in this paper, the samples come from financial texts or other texts). As mentioned earlier, for the feature extraction subnetwork, we hope to extract features that cannot distinguish the sample data domain so as to achieve the purpose of transfer learning. However, this purpose is contradictory to the purpose of domain classification. In order to solve this problem, as shown in Figure 4, a reverse gradient layer is added to the traditional classification network. The reverse gradient layer can be expressed as follows:
  (2) $$\begin{array}{c} RG\left(x\right)=x,\\ \frac{\partial RG\left(x\right)}{\partial x}=-\tau I. \end{array}$$

Among them, **RG**(.) represents the function of the forward propagation of the reverse gradient layer. During the forward propagation, this layer does not have any influence on the propagation of the data. When the gradient is backpropagated, the layer adds a minus sign to the original gradient. Designing the reverse gradient layer in this way can enable the domain classification subnetwork trainable and enable the feature extraction subnetwork to obtain features that cannot distinguish domains.

### 2.2. The Loss Function for the Proposed Network

According to the above descriptions, Table 1 shows the symbols of functions and parameters in different subnetworks.

According to the above symbols, it can be seen that there are two cost functions in the network structure:

(1) Cost function for sentiment classification: the cost can be expressed by *C*_*s*_, which can be written as follows:
  (3) $$\begin{array}{c} C_{s}\left(\theta_{f},\theta_{s}\right)=\sum_{i=1}^{M}C_{s,i}\left(F_{s}\left(F_{f}\left(x_{i};\theta_{f}\right);\theta_{s}\right),s_{i}\right), \end{array}$$
here *i* denotes the index of the sample, *M* represents the number of samples, *s*_*i*_ represents the sentiment label of the corresponding sample, **x**_*i*_ represents the sample of the input source domain, and *C*_*s*,*i*_(.) represents the corresponding cross-entropy function, which can be written as follows:
  (4) $$\begin{array}{c} C_{s,i}\left(.\right)=-\sum s_{i}\text{log}O_{i}, \end{array}$$
where *O*_*i*_ represents the output of the softmax layer and *s*_*i*_ represents the number of one-hot encoding based on the actual sentiment classification label.
(2) Domain classification cost function: the cost can be written as follows:
  (5) $$\begin{array}{c} C_{d}\left(\theta_{f},\theta_{d}\right)=\sum_{i=1}^{M}C_{d,i}\left(F_{d}\left(F_{f}\left(x_{i};\theta_{f}\right);\theta_{d}\right),d_{i}\right). \end{array}$$
Among them, *C*_*d*_ represents the cost function, *i* represents the index of the sample, *M* represents the number of samples, *d*_*i*_ represents the domain label of the corresponding sample, **x**_*i*_ represents the sample of the input source or target domain, and *C*_*d*,*i*_(.) represents the domain classification cross-entropy:
  (6) $$\begin{array}{c} C_{d,i}\left(.\right)=-\sum d_{i}\text{log}O_{i}, \end{array}$$
where *O*_*i*_ represents the output of the softmax layer of the domain classification subnetwork and *d*_*i*_ represents the number of domain sources after one-hot encoding.

According to the sentiment classification cost function and domain classification cost function, the overall cost function can be written as follows:

(7) $$\begin{array}{c} C_{all}\left(\theta_{f},\theta_{s},\theta_{d}\right)=C_{s}\left(.\right)-\tau C_{d}\left(.\right)\text{,} \end{array}$$

where *C*_all_ represents the overall cost function, and the parameter *τ* denotes the ratio of the two cost functions. Noting that here, a minus sign is added before the domain classification cost function, which is equivalent to the operation of the gradient backpropagation layer mentioned above. After the addition of the minus sign, the training process is equivalent to maximizing the cost of the domain classification subnetwork while minimizing the cost of the sentiment classification subnetwork. In this way, the features extracted by the feature extraction subnetwork can be both effective for distinguishing sentiment labels and at the same time insensitive for distinguishing domains.

### 2.3. The Training Processes

According to the overall cost function mentioned above, during training, different subnetwork parameters can be updated as follows:

(8) $$\begin{array}{c} \theta_{f}⟶\theta_{f}-\mu \left(\frac{\partial C_{s}}{\partial \theta_{f}}-\tau \frac{\partial C_{d}}{\partial \theta_{f}}\right),\\ \theta_{s}⟶\theta_{s}-\mu \frac{\partial C_{s}}{\partial \theta_{s}},\\ \theta_{d}⟶\theta_{d}-\mu \frac{\partial C_{d}}{\partial \theta_{d}}. \end{array}$$

The parameter *μ* indicates the update rate. The value of the learning rate *μ* in our implementation is set to 0.0005. The value is a typical value for the learning rate.

In actual training process, training is carried out in batches. In order to optimize all cost function items in each batch, the batch adopted here needs to include the samples of the source domain and the target domain. As a result, during optimization, there is a certain confrontational relationship between minimizing the cost of sentiment classification and maximizing the cost of domain classification. Here, the parameter *τ*_*p*_ determines the proportion of the two cost functions. During the training process, the hyperparameter *τ*_*p*_ is set according to the following equation:

(9) $$\begin{array}{c} \tau_{p}=\frac{2}{1+\text{exp}\left(-\alpha p\right)}-1. \end{array}$$

To set the hyperparameter according to the above formula, the advantages are as follows. (1) At the early stage of training, the recognition rate is low and the value of *p* is close to 0. Setting *p* close to 0 can make the training network become a pure sentiment classification network without considering the problem of transfer learning. (2) When *p* increases, *τ*_*p*_ also increases. It means that with the convergence of the feature extraction and sentiment classification subnetwork during training, the cost of transfer learning should be taken into consideration. The parameter of *α* determines the conversion speed for the two situations. In one situation, only the recognition rate is considered. In the other, only the domain classification accuracy is considered. In the above formula, the value of *α* is set to 25 according to subsequent experiments.

## 3. Results and Discussion

### 3.1. The Adopting Dataset

In order to verify the proposed cross-domain sentiment classification method, the classic Amazon dataset is adopted. The dataset contains more than 340,000 reviews, which cover 22 different products. Because the dataset contains too many types, the size of the set is large, and the positive and negative reviews are uneven; herein, the corresponding reduced Amazon dataset is adopted for experimentation. The basics of the reduced dataset are shown in Table 2. It can be seen that the dataset contains four different domains of texts: books, DVDs, electronics, and kitchen appliances. Each category contains 2000 tagged reviews, of which 1000 are positive reviews and 1000 are negative reviews, each accounting for 50%. In addition, they each have a few thousand unlabeled reviews. The reduced dataset can be adopted to evaluate the domain adaptation method proposed in this paper. In order to verify the effect of sentiment classification in the financial text field, the Microblog dataset in Stocktwits is also adopted, which contains 2000 unlabeled samples and 500 samples with labels. In this experiment, this dataset can be used as the target domain for research.

### 3.2. The Performance of the Proposed Method

According to the dataset mentioned above, the proposed method can be verified. The reviews of the four products in the reduced Amazon dataset are used as the source domains, and the Microblog dataset is used as the target domain for training and testing. The test results are shown in Table 3, and the different source and target domains corresponding to different situations in the table are shown in Table 4. It can be seen that the proposed method can effectively perform cross-domain sentiment classification, and its correction rate can reach 65.0%, 61.2%, 61.6%, and 66.3% under the situation of A, B, C, and D, respectively; compared with the results without transfer learning, the recognition rates have been improved by 11.0%, 7.6%, 11.4%, and 13.4%, respectively. For the average recognition rate, it is improved by 10.9%. The method without transfer learning is the method without the domain classification subnetwork. For this subsection, the main purpose is to validate the transfer learning method. Therefore, the method to compare has the same structure as the proposed method, where the only difference is that there is no domain classification subnetwork.

## 4. Method Comparisons

The proposed method is compared with the previously mentioned SVM-based method, the parameter fine-tuning method, and the SDA-based transfer method. Herein, the SVM-based method is used as the baseline method. Note that in the baseline method, transfer learning is not performed. The purpose of adding the baseline method is to evaluate the effect of the transfer learning method according to the following metrics. In order to better compare the transfer effect, inspired by [14], the transfer ratio index *R*_*t*_ and in-domain ratio index *R*_*i*_ are defined. Among them, the transfer ratio index *R*_*t*_ can be expressed as follows:

(10) $$\begin{array}{c} R_{t}=\frac{1}{N}\sum_{\left(s,t\right)}\frac{e\left(s,t\right)}{e_{b}\left(t,t\right)}, \end{array}$$

where *s* and *t* represent the source domain and the target domain respectively; *N* represents the number of pairs from the source domain to the target domain; *e*(*s*, *t*) represents the transfer error, which denotes the test error of transfer learning; and *e*_*b*_(*t*, *t*) represents the in-domain error according to the baseline method. The transfer ratio index reflects the transfer learning effect of the corresponding method, and its value is positively correlated with the performance of the transfer learning method. The in-domain ratio index can be expressed as follows:

(11) $$\begin{array}{c} R_{i}=\frac{1}{M}\sum_{\left(s\right)}\frac{e\left(t,t\right)}{e_{b}\left(t,t\right)}. \end{array}$$

**Figure 5:**
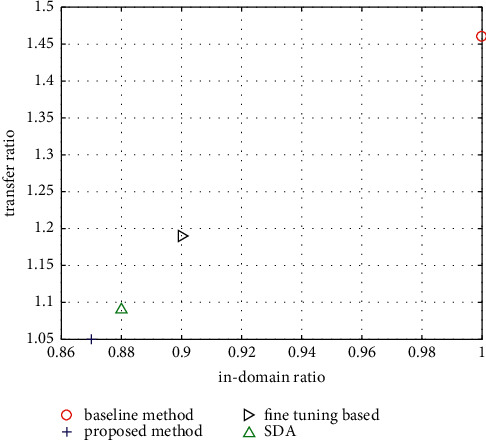
Comparisons of transfer ratio and in-domain ratio of different methods.

Among them, *M* represents the number of all domains; *e*(*t*, *t*) represents the in-domain error of the corresponding method, that is, the test error under the condition that both training and testing are in the source domain, and *e*_*b*_(*t*, *t*) represents the in-domain error of the baseline method. The in-domain error can be used to represent the sentiment classification effect of the method under the conditions of the same data domain for training and testing. The smaller the value is, the better the classification performance is.  Figure 5 shows the comparisons of the transfer ratio and in-domain ratio of the proposed method, the baseline method, the parameter fine-tuning method, and the SDA method. It can be seen that the values of transfer and in-domain errors of the proposed method in this paper are both the smallest, which shows that the proposed method has the best classification performance not only under the same domain but also under the condition of cross-domains. For the adopted dataset, the data volume is not large enough for the GAN-based network. Therefore, it is not compared in our implementation. This also shows the disadvantage of the GAN-based method, which is hard to be trained. The performance of the GAN-based method will be studied in the future.

**Figure 6:**
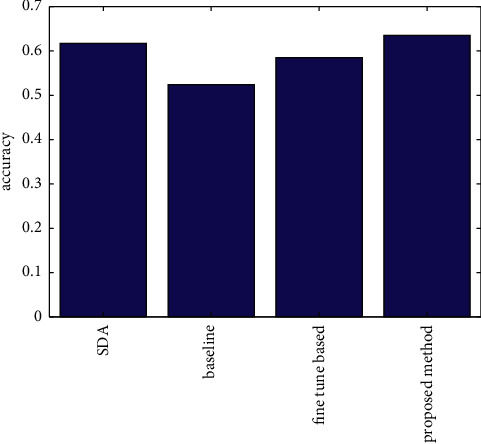
Bar plot of average recognition rate of different methods.

Figure 6 shows the bar plot of the cross-domain recognition rates, including the proposed method, the baseline method, the parameter fine-tuning method, and the SDA method.  Table 5 shows the comparisons of the cross-domain average recognition rate of the four methods. It can be seen that the cross-domain recognition rate of the proposed method has reached 63.5%. Compared with the baseline method, the parameter fine-tuning method, and the SDA method, the average recognition rate has improved by 11.1%, 5.1%, and 1.8%, respectively.

## 5. Conclusions

For financial-text-based sentiment classification, it is often difficult to obtain a high recognition rate due to the lack of labeled samples for financial texts. In order to solve the problem, a transfer learning method based on domain adaptation is proposed in this paper. In the proposed method, a large amount of source domain text samples is adopted for sentiment classification. The proposed method is a cross-domain transfer-learning-based method. The domain classification subnetwork is added to the original sentiment classification network, and the domain classification loss function is also added to the original training loss function. Therefore, the network can simultaneously adapt to the target domain and accomplish the classification task. The experiment of the proposed sentiment classification transfer learning method is carried out through the open-source dataset. The proposed method in this paper uses Amazon books, DVDs, electronics, and kitchen appliances as the source domain for cross-domain learning, and the classification accuracy rates have reached 65.0%, 61.2%, 61.6%, and 66.3%, respectively. Compared with nontransfer learning, the classification accuracy rate has improved by 11.0%, 7.6%, 11.4%, and 13.4%, respectively. Compared with the SVM-based baseline method, the CNN-based parameter fine-tuning transfer learning method, and the SDA-based method, the average recognition accuracy rate has been improved by 11.1%, 5.1%, and 1.8%, respectively.

For the adopted dataset, the data volume is not large enough for the GAN-based network. Therefore, it is not compared in our implementation. This also shows the disadvantage of the GAN-based method, which is hard to be trained. The performance of the GAN-based method will be studied in the future.

## Figures and Tables

**Table 1:** Symbols for the subnetworks.

Subnetwork	Network symbol	Parameter symbol
Feature extraction	*F*_*f*_(.)	*θ*_*f*_
Sentiment classification	*F*_*s*_(.)	*θ*_*s*_
Domain classification	*F*_*d*_(.)	*θ*_*d*_

**Table 2:** The basics of the reduced Amazon dataset.

Products	Labeled number	Unlabeled number
Books	2000	4465
DVDs	2000	3586
Electronics	2000	5681
Kitchen	2000	5945

**Table 3:** The test results of the proposed method.

Situation	Proposed (%)	Without transfer learning (%)
A	65.0	54.0
B	61.2	53.6
C	61.6	49.8
D	66.3	52.9

**Table 4:** Correspondences between source and target domains in different situations.

Situation	Source domain	Target domain
A	Books	Microblog
B	DVDs	Microblog
C	Electronics	Microblog
D	Kitchen	Microblog

**Table 5:** Comparisons of average recognition rate of different methods.

Method	Accuracy (%)	Improvement
Baseline	52.4	11.1%
Proposed method	62.5	—
Fine-tune-based	58.5	5.1%
SDA	61.7	1.8%

## Data Availability

The Amazon dataset is publicly available.
